# Supplementary material for: Memristor-based analogue computing for brain-inspired sound localization with in situ training
Source: Nat Commun. 2022 Apr 19;13:2026. doi: 10.1038/s41467-022-29712-8 (PMC9018844; doi:10.1038/s41467-022-29712-8)
Supplement: Supplementary file 1 — Supplementary Information [file 41467_2022_29712_MOESM1_ESM.pdf]

Memristor-Based Analogue Computing for Brain-Inspired Sound Localization with in situ training

Bin Gao[1, #, *], Ying Zhou[1, #], Qingtian Zhang[1, #], Shuanglin Zhang[1], Peng Yao[1], Yue Xi[1], Qi Liu[1], Meiran Zhao[1], Wenqiang Zhang[1], Zhengwu Liu[1], Xinyi Li[1], Jianshi Tang[1], He Qian[1], and Huaqiang Wu[1, *]

[1]School of Integrated Circuits, Beijing National Research Center for Information Science and Technology (BNRist), Tsinghua University, Beijing, 100084, China.

[#]These authors contributed equally to this work

[*]Email: gaob1@tsinghua.edu.cn; wuhq@tsinghua.edu.cn

This Supplementary Information includes:

Note 1. Principles of sound localization network

Note 2. The in-situ training schemes

Note 3. Simulation model

Note 4. Estimation of multi-thresholds-update schemes

Note 5. Measurement platform

Note 6. In-situ training results for comparison

Note 7. Parameter exploration

Note 8. Benchmarking of memristor-based sound localization for comparison

Supplementary Note 1

Principles of sound localization network

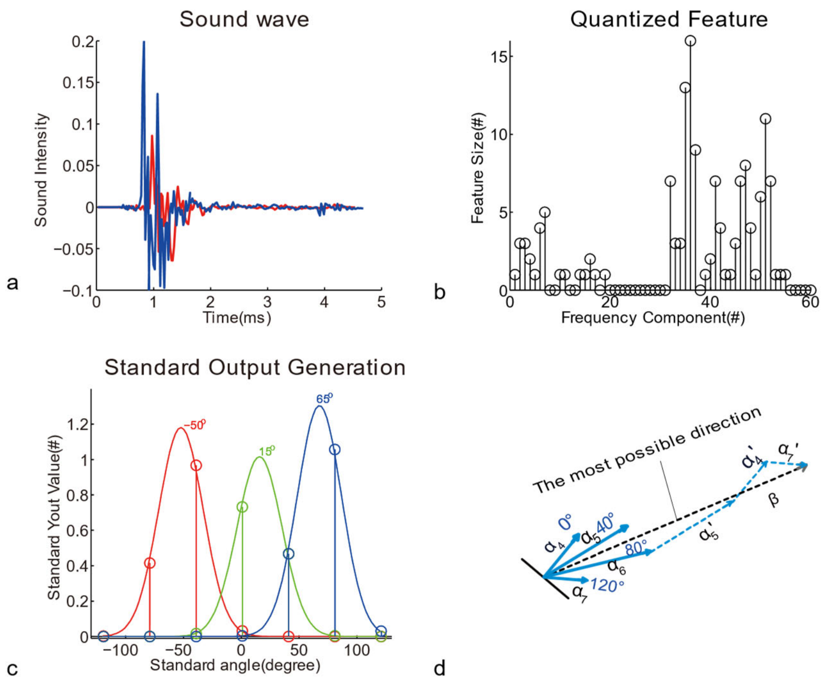

Supplementary Fig. 1. The input and output data of sound localization. **a.** Dual channel sound wave samples from the same sound source. **b.** Generated features to be used in the network. The sound signals are transformed to the frequency domain and then quantized to 16 levels as input features. **c.** Supervised output generation. It is described with Gauss functions (the full curves) and sampled every 40 degrees. **d.** Output values from the network are added together as vectors.

Supplementary Note 2

The in-situ training schemes

In-situ training might be more suitable for memristor-based analogue computations due to its better error tolerance. In the previous study, two schemes to update the weight value were adopted during the in-situ training process, namely, in-situ with verification and without verification, as shown in Supplementary Fig. 2. For the first case, with multiple pulses, the weight is programmed until the conductance reaches the desired weight. For the second case, one update pulse (SET/RESET) is chosen according to the sign of the calculated update value.

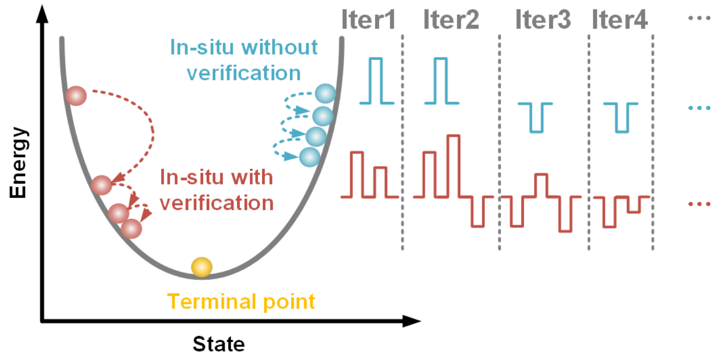

Supplementary Fig. 2. A comparison of the two in-situ weight update methods during the training process.

Supplementary Note 3

Simulation model

The device model used in the simulation is developed by fitting measured data based on a random walk model. G means the current conductance state. The conductance between G_{min} and G_{max} is divided into 10 intervals. The conductance is calculated by the following equation:

$$G' = G + (a + b \cdot \sigma_{wr}) \quad (1)$$

In this equation, a and b are fitting parameters in different intervals. G is the current conductance state. σ_{wr} is a random number related to the variance scale factor. G' is the next conductance value after applying a SET/RESET pulse. The fitting parameters a and b during SET/RESET operation are presented in Supplementary Fig. 3a and b. To show the effect of variance on the device characteristics more intuitively, the following figures illustrate the analogue behavior with different variance scale factors.

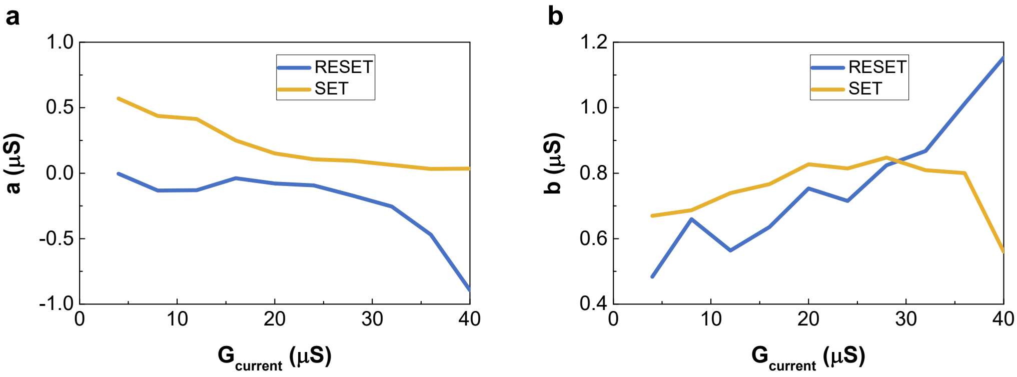

Supplementary Fig. 3. Fitting parameters in the memristor model.

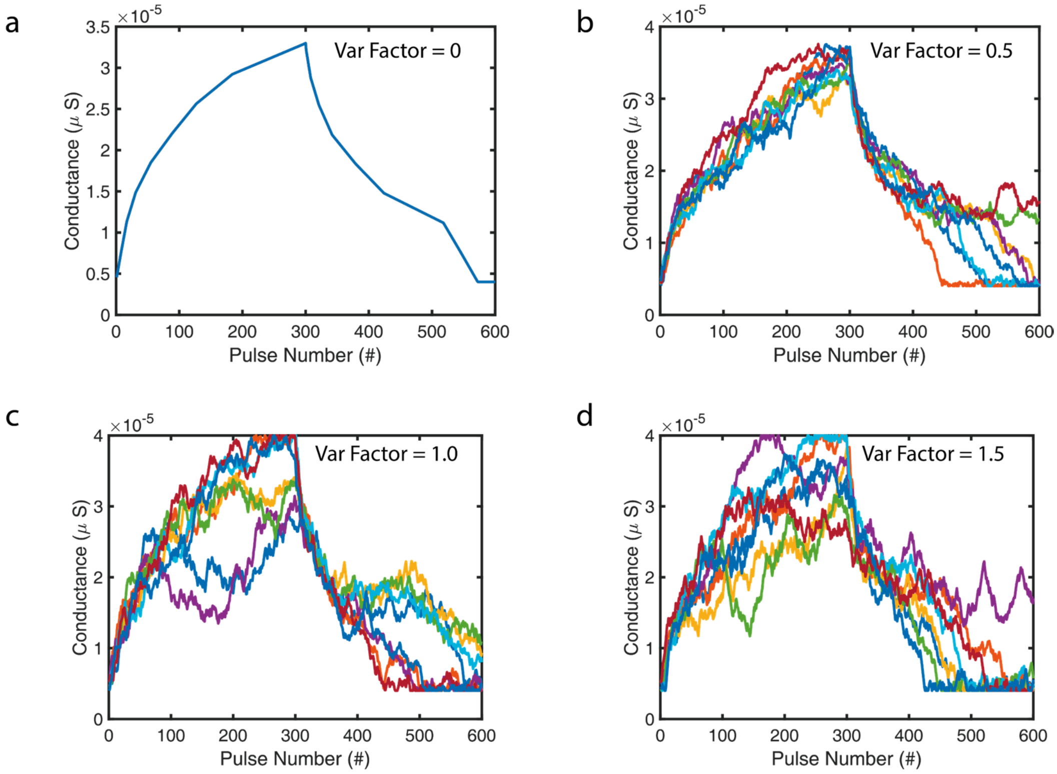

Supplementary Fig. 4. Simulated analogue behavior with variance factors of **a.** 0, **b.** 0.5, **c.** 1.0 and **d.**1.5. When the variance scale factor equals 1.0, it reflects the actual behavior of memristors.

Supplementary Note 4

Estimation of multi-thresholds-update schemes

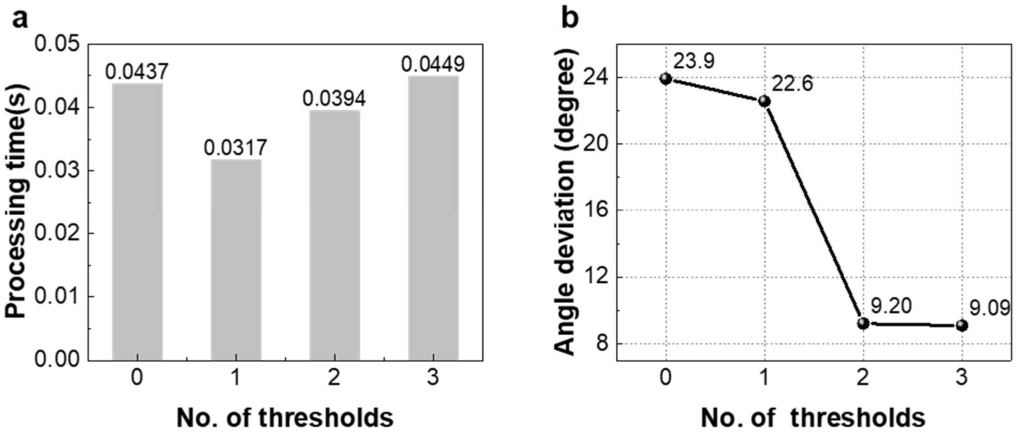

Supplementary Fig. 5. a. Processing time spent on the memristor array during in-situ training under various number of update thresholds. The programming pulse width is 50 ns. **b.** Training results of the two-layer sound localization network with different number of update thresholds. The number of input, hidden, output neurons are respectively 80, 40, 7. The output neurons contribute for the final prediction angle.

Supplementary Note 5

Measurement platform

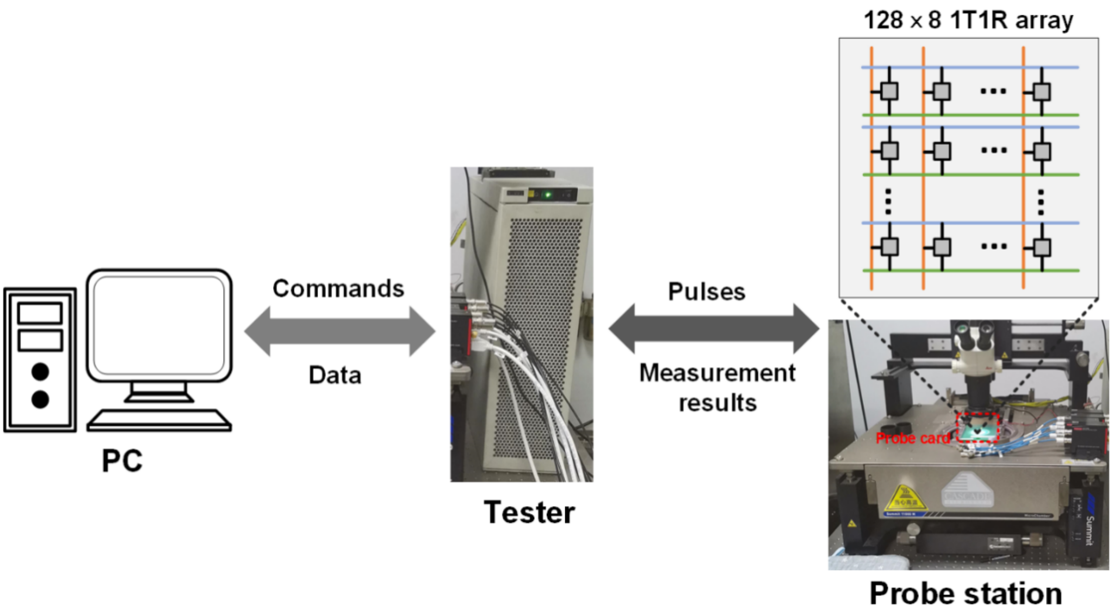

Supplementary Fig. 6. Schematic of the measurement platform, including PC, tester, and probe station (the probe card is connected to the pads of the memristor chip). The tester is the measurement equipment that is connected with the PC and probe card. During the training process, according to received commands, the tester generates SET/RESET/READ pulses and sends them to a probe card that is connected to the memristor array. The current flow through SLs is collected by the probe card and sent back to the tester. Then, the tester delivers data to the connected PC for further analysis.

Supplementary Note 6

In-situ training results for comparison

Situation	No. of thresholds=2	No. of thresholds=0	Ideal quantized network (W_{bit}=1, O_{bit}=3)	Ideal quantized network (W_{bit}=2, O_{bit}=3)
**Average normalized MSE of angle deviation**	0.0927	0.171	0.118	0.109

Supplementary Table 1. The average normalized mean square error (MSE) of the experimental results under the No. of thresholds = 0 and 2 and ideal quantized networks (including W_{bit} (weight precision) =1&2, O_{bit} (output precision) =3).

Supplementary Note 7

Parameter exploration

We investigate and simulate the in-situ training performance with asymmetry, linearity. Detailed results and discussions are shown below:

Asymmetry

Supplementary Fig. 7a illustrates memristor models with asymmetric and symmetric update behaviors. Using the multi-threshold-update scheme, training accuracy and hardware cost of sound localization network are presented in Supplementary Fig. 7b-d.

With the same zero-threshold-update scheme, the training effect with the asymmetric model is a little better than the symmetric device. For symmetric devices with low conductance values (see black line in Supplementary Fig. 7a), a SET/RESET pulse will lead to a large conductance change (large learning rate). During the in-situ training process, conductance of many key devices fluctuates in the low conductance range and hardly reaches to the target value. It differs from the training process with asymmetric device and causes a loss of training accuracy. In contrast, although the asymmetric devices with low conductance values have a large conductance change with a SET pulse, they can have a small conductance change with a RESET pulse, resulting in the improvement of accuracy. In this task, compared to initial state 4μS, random initialization is more conducive for the symmetric device model. Supplementary Fig. 7c-d present the hardware overhead of sound localization network with various in-situ training schemes, including processing time and energy consumption. Considering the hardware cost and training results, two-threshold-update-scheme is more appropriate.

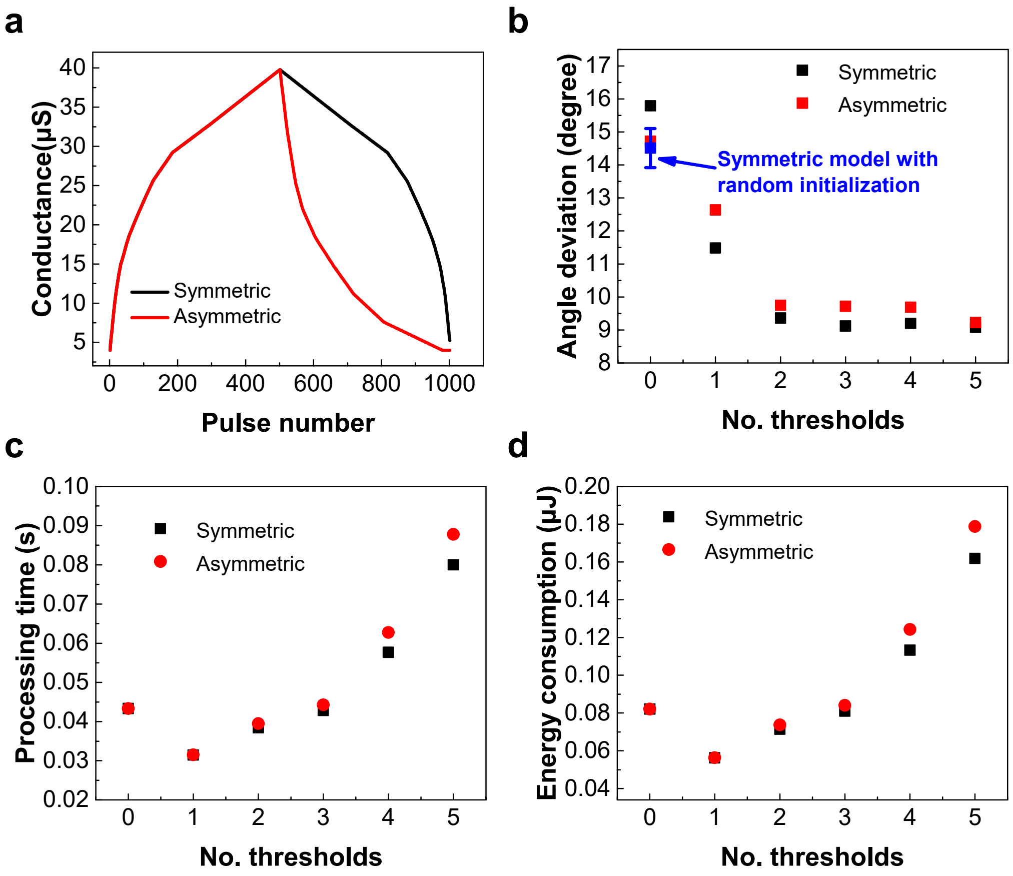

Supplementary Fig. 7 The impact of asymmetric behavior on the performance of memristor-based sound localization. **a.** The device model with asymmetric and symmetric behaviors. **b.** Training accuracy with various number of thresholds. **c.** Processing time spent on the memristor array during in-situ training process. **d.** Energy consumption spent on the memristor array during in-situ training process.

Nonlinearity

As illustrated in Supplementary Fig. 8, we simulate the training accuracy with linear and nonlinear device models. For different nonlinearity characteristics, different training schemes are adopted. The thresholds and pulse numbers are the best solutions currently explored for various models. It is worth noting that with the introduction of one update threshold, the nonlinear device shows a better training result compared to the linear device. For the nonlinear analogue behavior, with the low initial state, the increase of conductance is greater than that of the linear model. It exhibits a wider range of conductance, making a higher training accuracy.

When the number of thresholds is greater than 2, the training results of sound localization network will tend to be saturated. The linear model shows a slightly better

training effect than the nonlinear model. Supplementary Fig. 8c-d present the hardware cost for nonlinear and linear switching models with multi-thresholds-update scheme. Considering hardware complexity and training results, the introduction of two thresholds is the more conductive for different models.

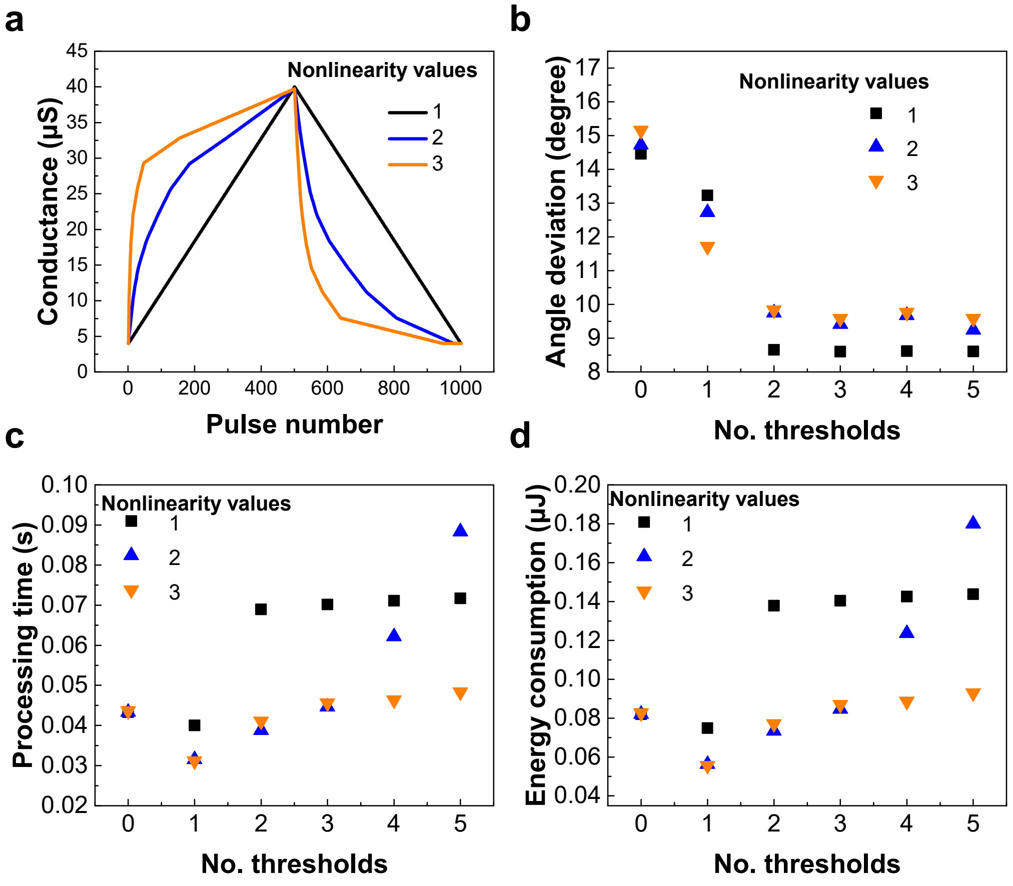

Supplementary Fig. 8 The impact of nonlinearity behavior on the performance of memristor-based sound localization. **a.** The device models with different behaviors. Curves 1,2,3 respectively reflect device models with increasing nonlinearity values. Model 2 is closer to the actual device. **b.** Simulated training accuracy with multi-thresholds-update scheme. **c.** Processing time spent on the memristor array under various number of update thresholds. **d.** Energy consumption spent on the memristor array under various number of update thresholds and device models.

Supplementary Note 8

Benchmarking of memristor-based sound localization for comparison

We evaluate the hardware cost of a larger memristor-based sound localization network with 200 input, 50 hidden and 11 output neurons. Output neurons contribute for the final detection results. As illustrated in Supplementary Table 2, the accuracy is comparable to the reported CMOS systems for the similar task[1-3]. As shown in Supplementary Fig. 9, the main circuit implementation for the sound localization network consists of a 2T2R memristor array, drivers, integrator, ADC and so on. The circuit design of angle vectors weighted summation is similar to the localization network, consisting of memristor cells, drivers, shift adder, ADC and so on. The outputs of the former layer are fed in the BLs as voltage pulses, and corresponding angles are mapped and stored as weights. Since the weight values could be positive or negative values, they are divided into $\mathbf{W}^+$ and $\mathbf{W}^-$. They are mapped to the upper and lower memristor devices in the 2T2R cell. In the 1-bit feed-forward process, the bit-line, BL^+ and BL^- are applied voltage pulse signals with opposite amplitudes according to the input value. V_{BL0+} to V_{BLN+} or V_{WL0} to V_{WLM} control the corresponding transmission gates, thereby affecting the voltage applied on the bit-line or word-line. The current flow through weight pairs is integrated by the corresponding source-line and compared with the reference voltage with a connected integrator and ADC[4,5]. The performance evaluation of modules during the feed-forward process for a sound sample is shown in Supplementary Fig. 10. The integrator and ADC contribute the most to the energy consumption. Supplementary Table 3 presents the overall performance of memristor-based sound localization. With small accuracy degradation, the memristor-based design respectively yields improvements of 184 × in the energy cost, and 3571 × in processing time compared to the CMOS design[1]. This work paves the way towards highly efficient all-analogue computing task.

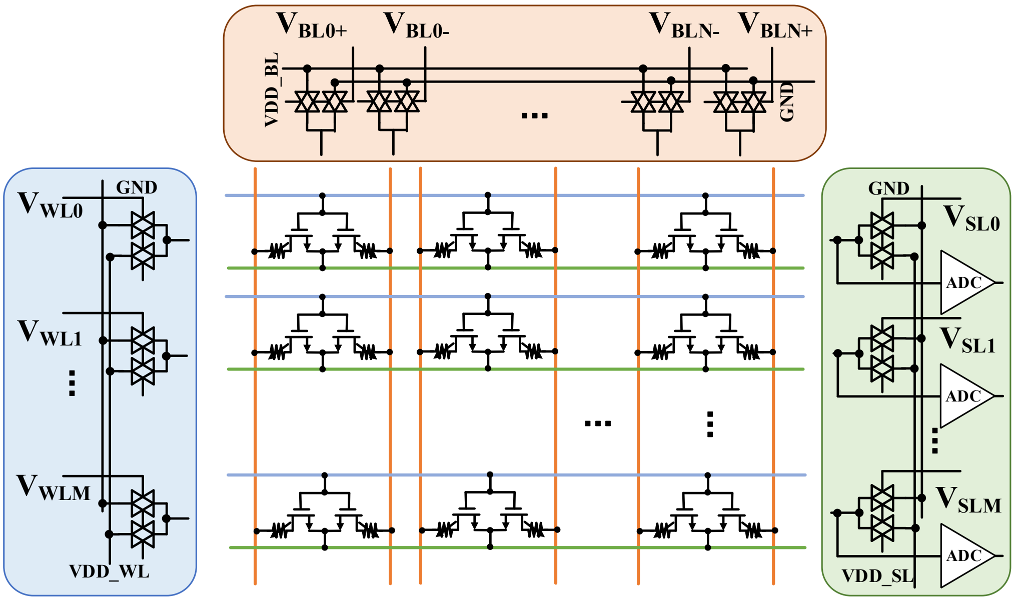

Supplementary Fig. 9. Schematic diagram of memristor-based sound localization, including the BL, WL, SL driver, integrator, ADC, and 2T2R (two-transistor-two-resistor) memristor array.

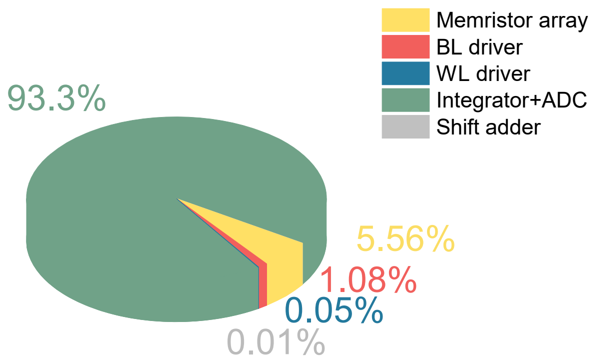

Supplementary Fig. 10. The energy consumption of modules during the feed-forward process for a sound sample.

Design	Hardware learning	RMSE (degrees)
**Memristor-based**	YES	12.5 (single-layer network, experiment) / 5.7 (two-layer network, simulation)
**DSLC system[1]**	NO	4.2
**Three-chip CMOS system[2]**	NO	5
**MEMS system[3]**	NO	8.7

Supplementary Table 2 The root mean square localization error (RMSE) results for single-layer and two-layer memristor network and comparisons with other designs, including dedicated sound localization

circuit (DSLC) system [1], a three-chip CMOS system[2], and a MEMS system[3].

Design	Technology node/nm	Processing time/ms	Energy consumption/μJ
**Memristor-based**	130	0.0028	0.306
**ASIC[1]**	130	10	56.3
**Improvement to ASIC**	-	3571×	184×

Supplementary Table 3 The benchmark results for memristor-based sound localization and comparisons with the sound localization chip (ASIC) in DSLC system. The memristor array and most of the peripheral circuits are evaluated with the simulator XPEsim[6]. The integrator and ADC data are obtained from the test and reference[4,5].

References

1 Jin, J. *et al.* Real-time Sound Localization Using Generalized Cross Correlation Based on 0.13 μm CMOS Process. *JSTS:Journal of Semiconductor Technology and Science*, doi:10.5573/JSTS.2014.14.2.175 (2014).

2 Grech, I. *et al.* Analog CMOS Chipset for a 2-D Sound Localization System. *Analog Integrated Circuits and Signal Processing* **41**, 167-184, doi:10.1023/B:ALOG.0000041634.92147.0d (2004).

3 Van Schaik, A. & Shamma, S. A Neuromorphic Sound Localizer for a Smart MEMS System. *Analog Integrated Circuits and Signal Processing* **39**, 267-273, doi:10.1023/B:ALOG.0000029662.37528.c7 (2004).

4 Paulus, C. *et al.* A 4GS/s 6b flash ADC in 0.13 /spl mu/m CMOS. *2004 Symposium on VLSI Circuits. Digest of Technical Papers (IEEE Cat. No.04CH37525)*, 420-423, doi:10.1109/VLSIC.2004.1346637 (2004).

5 Liu, Q. *et al.* A Fully Integrated Analog ReRAM Based 78.4TOPS/W Compute-In-Memory Chip with Fully Parallel MAC Computing. *2020 IEEE International Solid- State Circuits Conference - (ISSCC)*, 500-502, doi:10.1109/ISSCC19947.2020.9062953 (2020).

6 Zhang, W. *et al.* Design Guidelines of RRAM based Neural-Processing-Unit: A Joint Device-Circuit-Algorithm Analysis. *2019 design automation conference(DAC)* (2019).
