# Supplementary material for: Memristor-based analogue computing for brain-inspired sound localization with in situ training
Source: Nat Commun. 2022 Apr 19;13:2026. doi: 10.1038/s41467-022-29712-8 (PMC9018844; doi:10.1038/s41467-022-29712-8)
Supplement: Supplementary file 3 — Source data [file 41467_2022_29712_MOESM3_ESM.zip › Source file of Fig1a-d,Fig2a/Fig1a-c.pptx]

## Slide 1
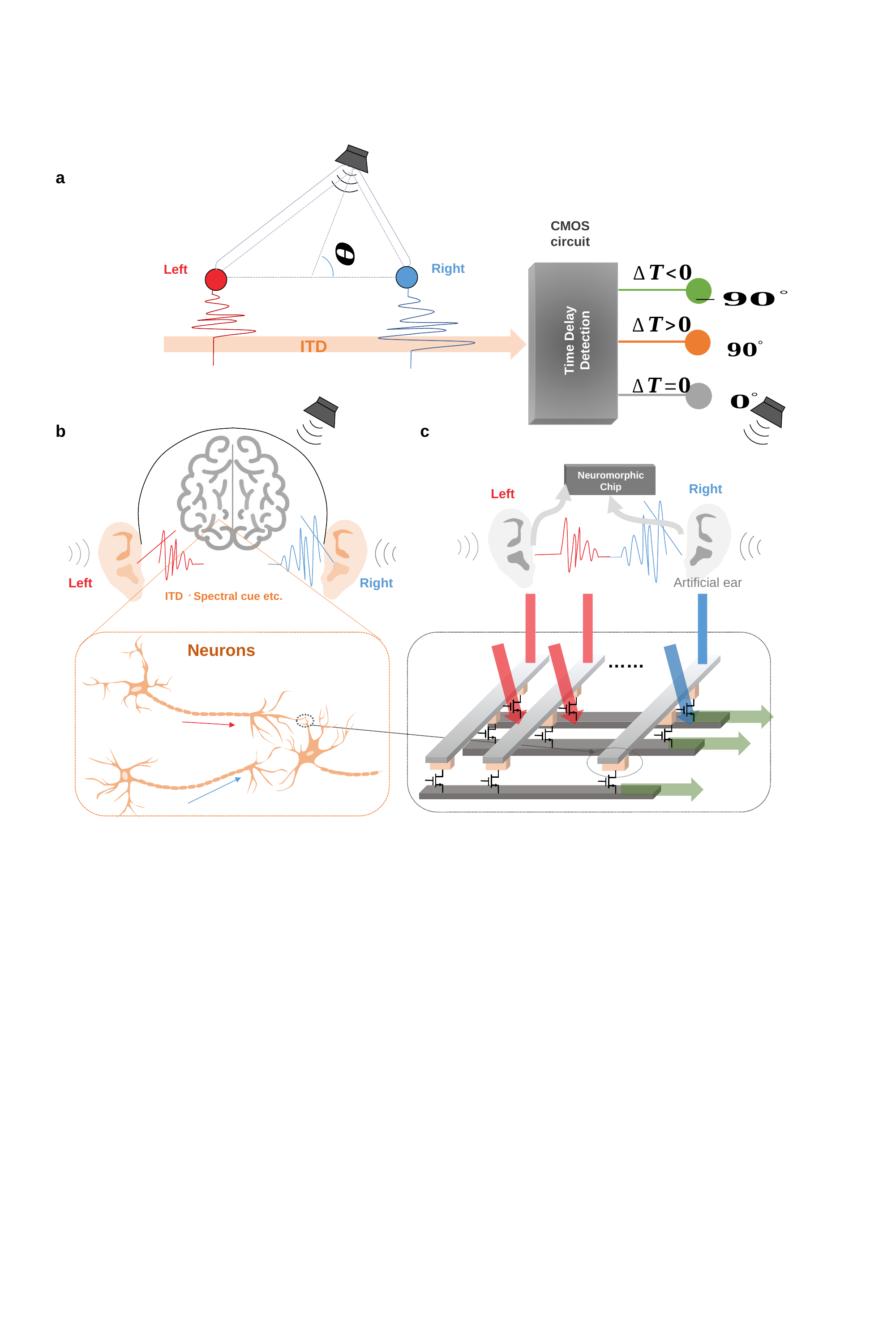

Right
Left
ITD
a
CMOS
circuit
Time Delay
Detection
b
c
Neuromorphic Chip
Right
Left
Artificial ear
Left
Right
ITD、Spectral cue etc.
Neurons
……
